# Mechanical and Optical Properties of Thermochromic Reversible Waterborne Primer Film on *Tilia europaea* with 1,2-Benzo-6-diethylaminofluorane Based Microcapsules

**DOI:** 10.3390/polym12092062

**Published:** 2020-09-10

**Authors:** Lin Wang, Xiaoxing Yan

**Affiliations:** 1 College of Furnishings and Industrial Design, Nanjing Forestry University, Nanjing 210037, China; wanglin@njfu.edu.cn; 2 Co-Innovation Center of Efficient Processing and Utilization of Forest Resources, Nanjing Forestry University, Nanjing 210037, China

**Keywords:** microcapsule, Tilia europaea, waterborne coating, reversible thermochromism, film performance

## Abstract

A waterborne thermochromic primer film containing thermochromic reversible microcapsules on the surface of Tilia europaea was prepared. The influences of different microcapsule concentrations on the reversible thermochromic, optical, mechanical and liquid resistance properties of the primer films were investigated. Not only were the morphology and structure of microcapsules and films observed, but also the chemical compositions of the microcapsules and films were analyzed. The results showed that for the primer film with microcapsules, the microcapsule concentration had a more significant influence on color difference. The primer film with microcapsules can achieve thermochromic reversibility. The temperature of color change was 32 °C and it had a good color recovery. The film gloss was negatively correlated with the microcapsule concentration, and the film with 5.0%–15.0% concentration had the best gloss. Adding an appropriate concentration of microcapsules can effectively improve the mechanical properties of the film. The film with 0–15.0% microcapsules had better liquid resistance to detergent, i.e., grade 1. The bonding form between the primer film added with microcapsules and Tilia europaea was physical bonding. This study provides a technical reference for the development of modern intelligent discoloration wood products.

## 1. Introduction

Microcapsules are a kind of micro container with core-shell structures, composed of dispersed solid, liquid or gas coated with film-forming materials [1]. The functionalization of microcapsules can be realized by coating different kinds of core materials [2]. At present, microcapsule technology is widely used in biomedicine [3], agriculture [4] and architecture [5]. As a kind of functional microcapsule, thermochromic reversible microcapsules have gradually become the focus of attention [6]. Thermochromic reversible microcapsules are a kind of material with intelligent response, which can change color with the ambient temperature changing [7]. That is, when the microcapsule is raised to a specific temperature, it can convert from its original color to another, and return to the original color with a drop in temperature [8].

Zhang et al. [9] successfully constructed two types of thermochromic phase-change microcapsules (TCMs) by preparing a silica-based wall filled with n-docosane core, afterwards forming a thermochromic indicator layer and polymer protective layer. These microcapsules had not only excellent heat-storage/release capabilities, but also good shape stability, high thermostability and good phase transition invertibility and endurance. Dong et al. [10] prepared reversible thermochromic paper with anticounterfeiting performance by adding reversible thermochromic microcapsules (RTM) into cellulose fiber slurry, which was difficult to simulate. It showed sharp color contrast under cool and heated conditions and offered unique anticounterfeiting features to the paper. Liu et al. [11] developed a nontoxic chlorophenol red (CPR) water thermochromic system and its microcapsule with silicone wall. The fabricated microcapsule exhibited visible discoloration and good invertibility, which could be applied as screen printing ink and a film additive. Li et al. [12] injected a methyl methacrylate dispersion containing VO_2_(M) @ SiO_2_ core-wall nanocomposites and thermochromic microcapsules into wood templates to prepare energy-saving wood composite with the outstanding optical, mechanical and dimensional stabilization properties. These studies show that thermochromic microcapsules can be used in latent heat-storage/release, anticounterfeiting, screen printing and energy saving. Thermochromic reversible microcapsules can be used as a new functional unit in the field of wood materials, bringing new opportunities for the development of wood-based functional materials.

The application of microcapsules to wood can generally be carried out through the impregnation treatment of microcapsule suspension into the pores of the wood [13], the physical mixing of microcapsules and adhesives into human-made boards [14], and the addition of microcapsules to the coating in advance through coating treatment [15]. But from the perspective of industrial production and “dynamic” color change decoration, the use of impregnation and adhesive may lead to problems such as high cost and large energy requirements, as well as uncertainty as to whether the microcapsules will maintain their shape and properties under heating and compressive stress [16]. Wood usually needs surface treatment prior to use [17]; the discoloration characteristics of wood materials depend mostly on the surface. Waterborne coatings mainly use water as a solvent or dispersant, which can effectively block the air pollution of traditional wood coatings, minimize the damage to human body caused by the coating, and have low construction requirements, which is favored by the majority of consumers [18]. However, there are few studies on the mechanical and optical performance of waterborne coatings with thermochromic microcapsules on the surface of wood. The 1,2-benzo-6-diethylaminofluorane-based microcapsules are based on the three-component system of the core materials of 1,2-benzo-6-diethylaminofluorane (dye), chromogenic agent and solvent, which are wrapped by melamine formaldehyde resin (wall material). The three-component system can ensure the thermochromism of the microcapsules and primer film added with microcapsules. If 1,2-benzo-6-diethylaminofluorane is added into the primer directly, the thermochromism will not occur. When 1,2-benzo-6-diethylaminofluorane is added with a suitable chromogenic agent and solvent to form a three-component system, discoloration can occur at about 30–32 °C; 1,2-benzo-6-diethylaminofluorane has high sensitivity and stable color change performance [19], which meets the requirements of wood color change.

In a previous work [20], a kind of waterborne thermochromic surface topcoat with thermochromic ink was studied on the surface of Cunninghamia lanceolata, which can change from red to colorless. For wood products, a single color no longer meets the needs of the public. The microcapsules studied in this paper can change from yellow to colorless. In this paper, a reversible thermochromic microcapsule which changes from yellow to colorless was selected as the functional unit, and Tilia europaea was selected as the base material. Microcapsules were added to the waterborne primer in advance; after that, they were coated on the base material surface to prepare a reversible thermochromic waterborne primer film. The effects of the concentration of the microcapsules on the performances of the reversible thermochromic waterborne wood primer film were investigated. This study promotes the development and application of reversible thermochromic intelligent response wood products composites, which lays the foundation for the realization of the combination of red and yellow colors.

## 2. Materials and Methods

### 2.1. Experimental Materials

Microcapsules were obtained from Oriental Color Technology Co., Ltd., Shenzhen, China. The main components of microcapsules were melamine formaldehyde resin (as wall material, CAS No. 9003-08-01), 1,2-benzo-6-diethylaminofluorane (Red DCF, dye, C_28_H_23_NO_3_, *M*_W_: 421.49 g/mol, CAS No. 26628-47-7), methyl palmitate (as solvent, C_17_H_34_O_2_, *M*_W_: 270.45 g/mol, CAS No. 112-39-0), ethyl stearate (as solvent, C_20_H_40_O_2_, *M*_W_: 312.5304 g/mol, CAS No. 111-61-5), styrene maleic anhydride monomethyl-maleate polymer (as emulsifier, C_17_H_16_O_7_, *M*_W_: 332.305 g/mol, CAS No. 31959-78-1). Dulux Muyun Jingwei antiscratch wood varnish (primer) was obtained from Keyuan Industrial Co., Ltd., Shanghai, China. The main components included waterborne acrylic copolymer dispersion, matting agent, additive and water. Tilia europaea, 100 mm × 65 mm × 4 mm, was provided by the laboratory of Nanjing Forestry University, Nanjing, China. The reagents used in the experiment were not further treated.

### 2.2. Fabrication of Coatings

**Figure 1:**
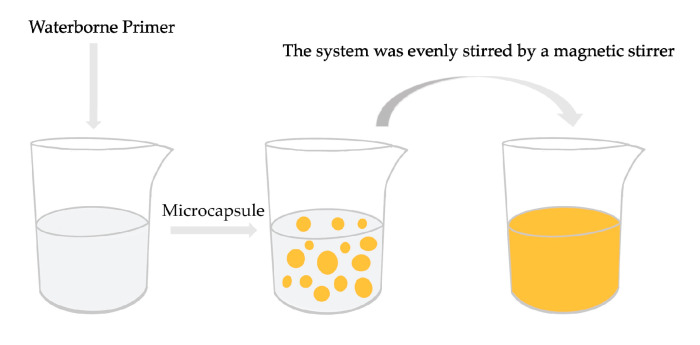
Fabrication process of waterborne coating added with thermochromic microcapsules.

**Figure 2:**
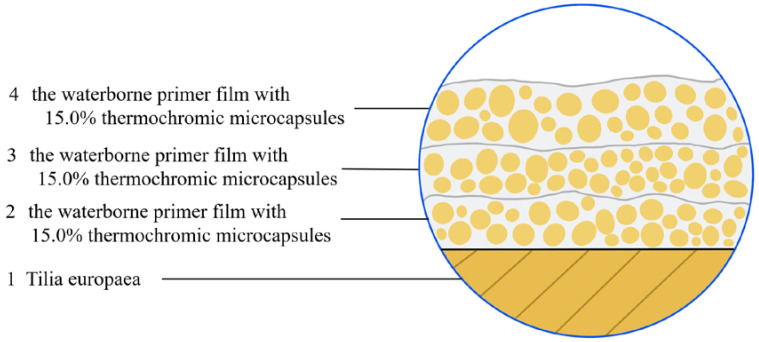
A Schematic diagram of sample 4#.

Figure 1 represents the fabrication process of the waterborne coating added with thermochromic microcapsules. The fabrication method of other samples was the same as Figure 2. In Table 1, the concentration of thermochromic microcapsules, drying temperature and drying time were selected as the experimental factors. The corresponding materials are shown in samples 1#–4# in Table 2. Samples 5#–11# in Table 2 were optimized experiments on the orthogonal basis. The 0–30.0 g thermochromic microcapsules and the 70.0–100.0 g primer were weighed, evenly mixed and coated on Tilia europaea with SZQ tetrahedral fabricator (Zhentong Trading Co., Ltd., Chengdu, China). Then, the sample was transferred to a 35 °C drying oven and heated for 20 min until the surface was dry, before being removed and allowed to cool to room temperature. The surface was softly polished with 800 mesh fine sandpaper, and afterwards, the floating powder was wiped off with dry cloth. The coating process was repeated twice. This sample after coating was moved to the drying oven again and dried at 35 °C for 2 h. After natural cooling, sample 1# was obtained. The preparation methods of other samples were identical to those of sample 1#. The thickness of the fabricated dried film approached 60 µm. The finished samples could be used for performance testing after 7 days’ storage at constant temperature and humidity (temperature 25 °C, relative humidity 77%).

### 2.3. Testing and Characterization

#### 2.3.1. Temperature Test

The test temperature range was 16–40 °C. Samples were heated using an HHP1 heating plate (Shanghai Hengyue Medical Devices Co., Ltd., Shanghai, China). Simultaneously, the temperature change of film surface was tested with a hand-held infrared thermometer (Taizhou Taiertan Automation Technology Co., Ltd., Jiangsu, China). The chromatic parameters of film from 16 °C to 40 °C and cooling from 40 °C to 16 °C were tested using SEGT-J Portable Colorimeter (Guoti Precision Testing Instrument Co., Ltd., Shenyang, China), and color difference was calculated. L* signifies lightness, a large value signifies that the chromaticity of the film surface is brighter, and a small value signifies the chromaticity of the surface is darker. a* signifies the chromaticity changes from red to green, a positive value signifies that the chromaticity is red, and a negative value signifies that the chromaticity is green. b* signifies the chromaticity changes from yellow to blue, a positive value signifies that the surface chromaticity is yellow, and a negative value signifies blue. C* is the chroma saturation. H* is the hue. The larger the value of *L*, the brighter the film; the higher the value of *a* is, the more red the film; and the greater the value of *b*, the more yellow the film. Based on the chromatic parameters of samples at 16 °C, the changes of chromatic parameters at different test temperatures during the heating and cooling experiments were recorded, and the variation tendency of the color difference (Δ*E*) of the samples at different temperatures during the heating and cooling process was calculated according to Hunter’s color difference Equation (1):

Δ*E* = [(Δ*L*)^2^ + (Δ*a*)^2^ + (Δ*b*)^2^]^1/2^ (1)

where Δ*L* (brightness difference) = L* − L*′, Δ*a* (red-green difference) = a* − a*′, Δ*b* (yellow-blue difference) = b* − b*′. Moreover, L*′, a*′, and b*′ represent the chromatic value of the film at 16 °C. *L*_1_, *a*_1_, and *b*_1_ represent the chromatic value of the film at other temperatures. In this test, 60° gloss of film was examined with a HG268 gloss meter (3NH Technology Co., Ltd., Shenzhen, China).

#### 2.3.2. The Hardness Test

The hardness was tested using a film hardness tester with a pencil (Biao Geda Precision Instrument Co., Ltd., Guangzhou, China). When testing the hardness, the angle between the pencil and the film was 45°, and the pencil was scraped under a 1.0 kg load. The hardness (measured with 6H–6B pencils) was tested once scratches appeared on the films. The pencil hardness signified the film hardness.

#### 2.3.3. The Adhesion Test

The film adhesion was tested using a QFH-HG600 film grader (Sunno Instrument Technology Co., Ltd., Tianjin, China). The handle of the ruler was held so that the multiblade cutter was perpendicular to the plane of film. The film was cut at a rate of 20–50 mm/s. The primer film was rotated 90°, and the step was repeated on the cut to make a grid pattern. The tape was stuck on the entire grid; afterwards, it was torn off at a small angle. The result could be obtained according to the proportion of the area of the film that was glued off. Grade 0 of the film adhesion indicates that the film adhesion was the best.

#### 2.3.4. The Impact Resistance Test

Impact resistance was tested using a QCJ-50 impactor (Yaoyang Instrument Equipment Co., Ltd., Cangzhou, China). A weight hammer was fixed at the required height by a controller screw, and its height could be read by the positioning mark. The controller screw was pressed to make the connected heavy hammer fall and act on the template previously placed on the pillow block. Then, the hammer body was lifted and taken out of the tested sample plate to observe the paint film on the sample plate. The impact strength of paint film was expressed by the maximum height at which the paint film was impacted by a 1.0 kg heavy hammer without causing damage. The unit of the impact resistance was kg·cm.

#### 2.3.5. The Liquid Resistance Test

The film liquid resistance was determined using 15.0% NaCl solution (Langfang Nabo Chemical Technology Co., Ltd., Langfang, China), 70.0% medical ethanol (Qingdao Haishi Hainuo Co., Ltd., Qingdao, China), detergent (containing 25.0% fatty alcohol ethylene oxide and 75.0% water, Hutchison Whitecat Co., Ltd., Shanghai, China), and red ink (Wenpai Trading Co., Ltd., Shanghai, China). During the experiment, filter paper was put into the test solution and soaked for 30 s. Then, it was picked up using a tweezer and put quickly onto the experiment area. The flowing liquid was wiped off and the sample was immediately covered with a toughened glass cover. The glass cover and the filter paper were moved 24 h later. The residual liquid on the surface of the film was absorbed by absorbent paper, and the damage, such as marks and discoloration of the sample area, was examined.

#### 2.3.6. Microstructure Test

The morphology of thermochromic microcapsules and films was analyzed using an Axio Scope A1 optical microscope (OM) (Carl Zeiss company, Jena, Germany) and Quanta 200 environment scanning electron microscope (SEM) (FEI company, Hillsboro, OR, USA). The particle size distribution of microcapsules was calculated according to the size of microcapsules in the SEM images. During the SEM test, the size of microcapsules was marked, and the microcapsules in the SEM images were selected for statistics. According to the percentage of the number of microcapsules within a certain size range and the total number of microcapsules, the particle size distribution of microcapsules was obtained.

#### 2.3.7. Infrared Spectrum Test

The component parts of the thermochromic microcapsules and films were analyzed with a vertex 80V infrared spectrum analyzer (Germany Bruker Co., Ltd., Karlsruhe, Germany).

All the experiments were repeated four times with an error of less than 5.0%.

## 3. Results and Discussion

### 3.1. Analysis of Morphology and Properties of Thermochromic Microcapsules

**Figure 3:**
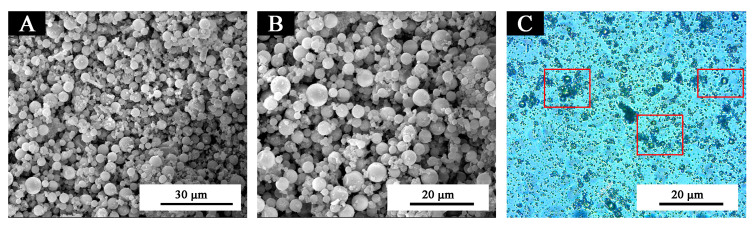
Morphology of thermochromic microcapsules: SEM of (**A**) low magnification and (**B**) high magnification, (**C**) OM of thermochromic microcapsules with light diffraction ring phenomenon.

**Figure 4:**
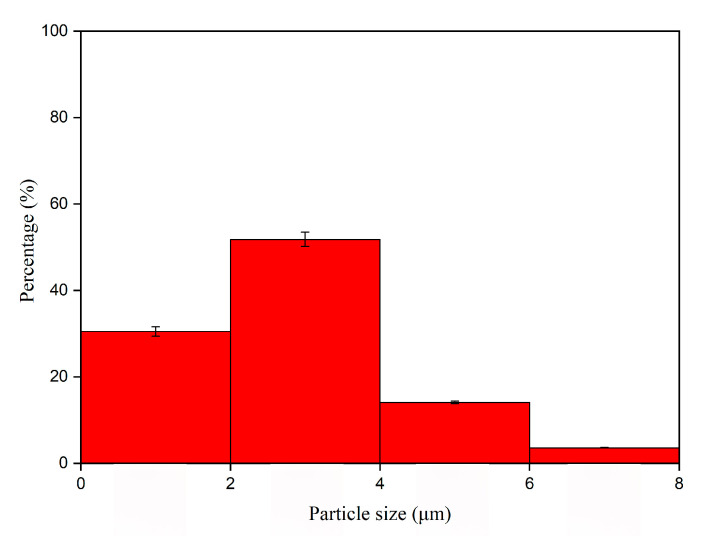
Particle size distribution of microcapsules.

The morphology of the thermochromic microcapsules is shown in Figure 3. The microcapsule particles were uniform and round, with a regular spherical shape. The surface of microcapsules was smooth and dense, and there was no agglomeration between microcapsules. According to Figure 3, the particle size of the microcapsules was noted; the distribution is shown in Figure 4. In Figure 4, the particle size distribution further shows that the microcapsules had uniform particle size and narrow distribution, and that the proportion of microcapsules with 2–4 µm was the highest, which was an ideal microcapsule. This was very beneficial to the orderly arrangement of microcapsules and the reflection of light. According to the principle that light propagates in the medium with unequal diffractive rate, a diffraction ring should be produced at the interface between the medium [21]. As shown in Figure 3c of OM diagram, the phenomenon of a light diffraction ring appeared in the thermochromic microcapsule, which indicated that there were two different media present. The dark outer ring represented the wall material and the transparent inner ring represented the core material. Moreover, the microcapsules were nearly circular, forming a clear core-wall structure, and the microcapsules were single core and single wall.

**Figure 5:**
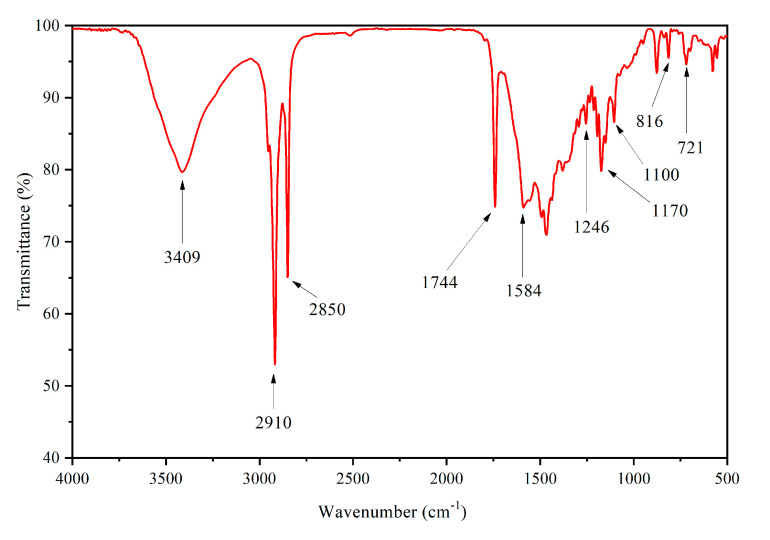
FTIR of thermochromic microcapsules.

The infrared spectrum of microcapsules is shown in Figure 5. Broad and strong absorption appeared at 3409 cm^−1^, which was caused by the superposition of stretching vibration absorption of –NH and –OH [22]. The stretching vibration absorption and bending vibration absorption of triazine ring were at 1584 cm^−1^ along with 816 cm^−1^, respectively [23]. At 1170 cm^−1^, 1100 cm^−1^ along with 1246 cm^−1^, stretching vibration absorption of aromatic ether C–O–C appeared [24]. The stretching vibration of carbonyl group in the conjugate chromophore structure appeared at 1744 cm^−1^, which indicated that 1,2-benzo-6-diethylaminofluorane existed in conjugate chromophore structure at low temperature [25]. The stretching vibration of –CH_3_ and –CH_2_ was observed at 2910 cm^−1^ along with 2850 cm^−1^ [26]. The in-plane bending vibration of –CH_2_ was observed at 721 cm^−1^ [27]. The infrared spectrum showed that the wall of microcapsules was melamine formaldehyde resin, and the core was a mixture of dye, chromogenic agent and solvent.

**Figure 6:**
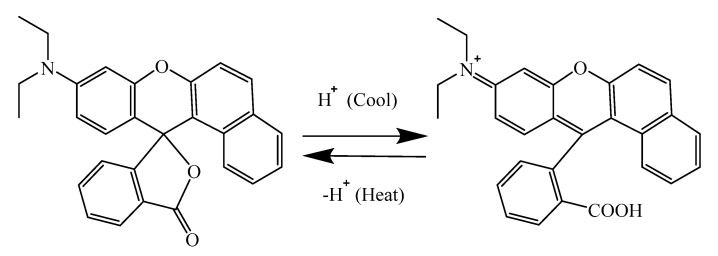
Discoloration principle of microcapsules.

**Figure 7:**
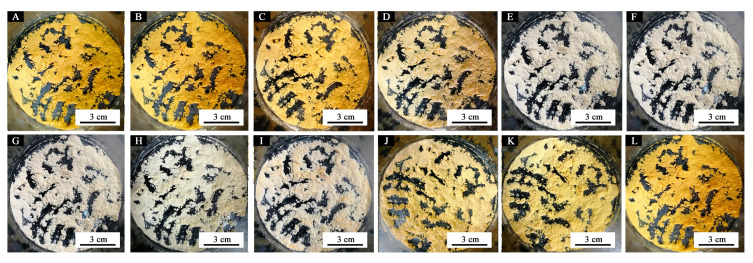
Color trend of thermochromic microcapsules changes with temperature: temperature increases to (**A**) 16 °C; (**B**) 26 °C; (**C**) 28 °C; (**D**) 30 °C; (**E**) 32 °C; (**F**) 40 °C; temperature drops to (**G**) 40 °C; (**H**) 32 °C; (**I**) 30 °C; (**J**) 28 °C; (**K**) 26 °C; (**L**) 16 °C.

The compound 1,2-benzo-6-diethylaminofluorane is an electron donor dye with a lactone ring structure. The solid-liquid state of the solvent in the thermochromic microcapsules was controlled by the temperature increase and drop. Figure 6 shows the principle of the reversible thermochromism microcapsules. At the case of a temperature lower than the melting point of the solvent, 1,2-benzo-6-diethylaminofluorane reacted with acid chromogenic agents, resulting in electron transfer. The lactone ring of 1,2-benzo-6-diethylaminofluorane was opened to form a conjugated chromogenic structure. The central carbon atom changed from sp^3^ hybrid state to sp^2^ hybrid plane configuration, which made the microcapsule system yellow. When the temperature of the system rose, the solvent became liquid, and the 1,2-benzo-6-diethylaminofluorane was isolated from the chromogenic reagent. So, the quinone structure changed into lactone ring structure in the liquid state and did not develop in color [28]. This process was reversible, which gave rise to a reversible thermochromic function of the waterborne primer film on Tilia europaea. Figure 7a–d further confirmed that when the temperature range was 16–30 °C, the microcapsules were yellow. When the temperature was greater than or equal to 32 °C, the microcapsules were colorless. When the temperature increased or dropped, the color of microcapsules quickly responded. When the temperature dropped, the microcapsules returned to their original yellow color.

### 3.2. Orthogonal Experiment Analysis

**Figure 8:**
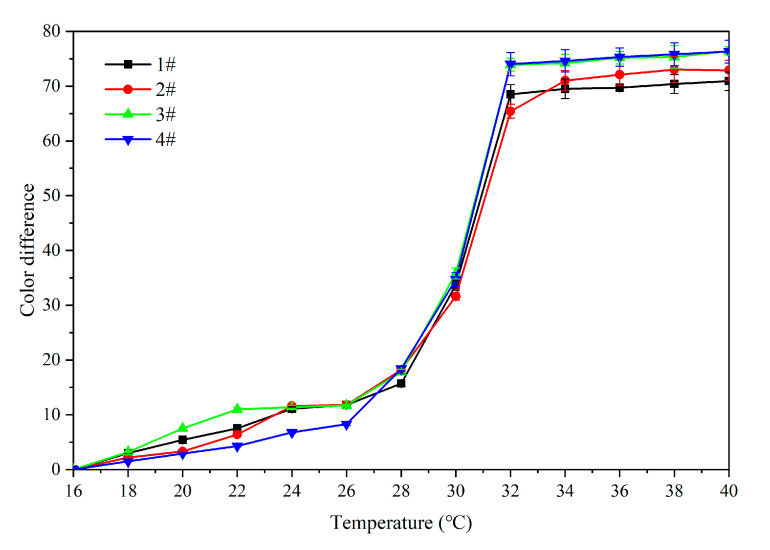
Effect of temperature increase (16–40 °C) on color difference of primer film added with microcapsules.

As shown in Figure 8, when the temperature increased from 16 °C to 28 °C, the color of the samples did not change significantly. As the temperature increased from 28 °C to 30 °C, the color began to change, showing a slow upward trend, albeit a minor one. The color difference basically reached the maximum value when the temperature reached 32 °C, which showed that the color change temperature range of samples 1#–4# was 30–32 °C, and that they yielded a thermochromic effect at this temperature.

The color difference results of the orthogonal test (Table 3) and the range showed that the concentration of microcapsules was the foremost factor affecting the color difference of the film, followed by drying temperature and drying time. According to the mean value of 1 and 2, it was further shown that the color difference of the primer film was more affected when the drying temperature was 35 °C and drying time was 2 h. Therefore, the influence of the microcapsule concentration on the reversible thermochromic property of primer film was determined.

### 3.3. Optimization of Experimental Analysis

#### 3.3.1. Effect of Microcapsule Concentration on Color Difference

**Figure 9:**
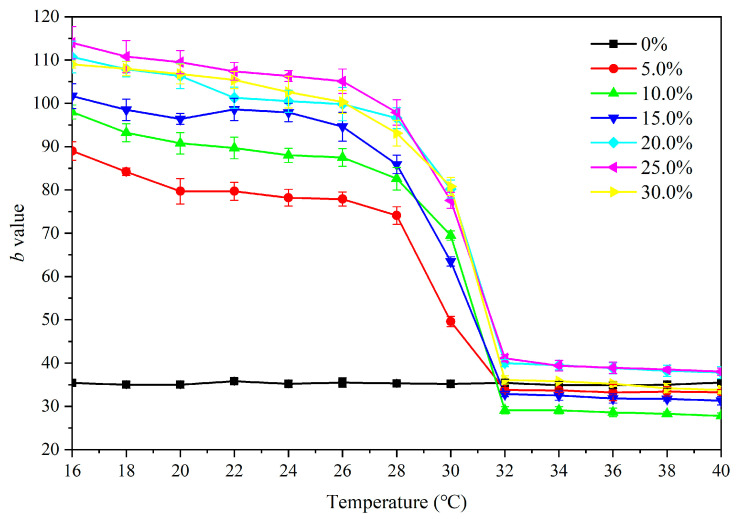
Effect of temperature increase (16–40 °C) on the *b* value of primer film added with thermochromic microcapsules.

As shown in Figure 9, with an increasing test temperature, the chroma parameter *b* value of the primer film without microcapsules was maintained at 34.0–36.0; the larger the *b* value, the more yellow the film color. A decreasing *b* value also indicated that the film color was changing from yellow to colorless step by step. The *b* value of the primer film did not change significantly from 16–28 °C at a 5.0–30.0% concentration. The *b* value of primer at 30 °C began to decline, but it did not change very much. As the temperature further increased to 32 °C, the *b* value of the film changed significantly and tended to be stable in the range of 32–40 °C.

**Figure 10:**
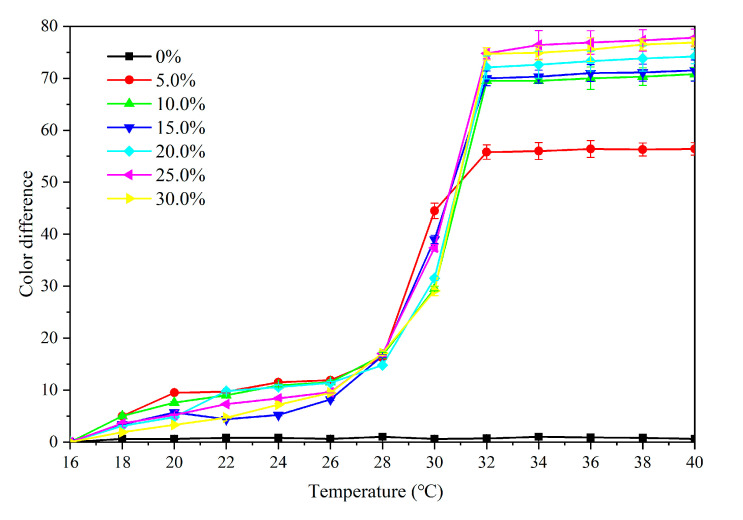
Effect of temperature increase (16–40 °C) on color difference of primer film with microcapsules.

**Figure 11:**
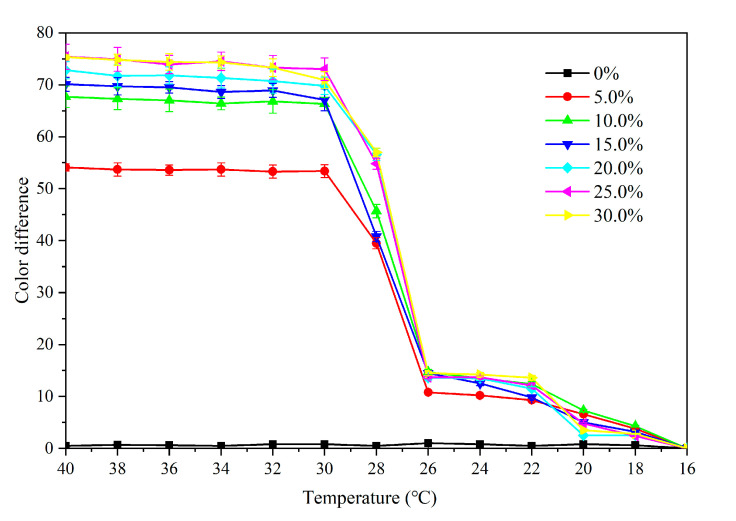
Effect of temperature drop (40–16 °C) on color difference of primer film with microcapsules.

**Figure 12:**
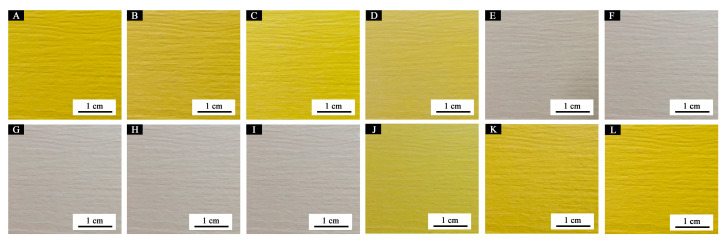
Color trend of primer film containing 15.0% microcapsules: temperature increases to (**A**) 16 °C; (**B**) 26 °C; (**C**) 28 °C; (**D**) 30 °C; (**E**) 32 °C; (**F**) 40 °C during the heating process; temperature drops to (**G**) 40 °C; (**H**) 32 °C; (**I**) 30 °C; (**J**) 28 °C; (**K**) 26°C; (**L**) 16 °C in the cooling process.

Figure 10 shows the color difference of the primer film from 16 °C to 40 °C. When the concentration of the microcapsules was 5.0–30.0%, the color difference was below 20.0 at 16–28 °C, and the thermochromic effect was not obvious. When the temperature increased to 30 °C, the color difference increased noticably. As the temperature increased to 32 °C, the color difference essentially reached the maximum value, i.e., the primer film changed color at that temperature. The color difference of the film without microcapsules was between 0–1.0, and there was no thermochromic effect. Moreover, the color difference of the film containing 5.0% microcapsules was smaller than that of the film with other concentrations, e.g., the thermochromic effect of the film with 10.0–30.0% microcapsule was superior. According to the color difference trend during the increasing and dropping temperature period (Figure 10 and Figure 11), the prepared primer film yielded a reversible thermochromic effect. When the microcapsule concentration was lower or higher than 15.0%, the color change trend of primer film was the same as that of 15.0% microcapsules. Therefore, 15.0% microcapsules was selected as an example. In Figure 12, at the 32 °C test temperature, the film color changed from yellow to colorless, and the original texture of wood was shown. When the test temperature dropped, the color of the primer film changed back to the original yellow, which proved that the film color could change, and that the thermochromic temperature range was 30–32 °C.

#### 3.3.2. Effect of Microcapsule Concentration on Gloss

The primer film gloss was measured with different light incidence angles (i.e., 20°, 60° and 85°). It can be seen from Table 4 that the addition of microcapsules had a great influence on the gloss of the film surface. At the same concentration of microcapsules, the film gloss increased with an increase of incident angle. At the same incident angle, the gloss of film containing 5.0% microcapsules was the highest. As the concentration exceeded 5.0%, the film gloss decreased. The reason for this was that when the concentration of microcapsules increased to a certain level, the surface film roughness increased, which resulted in light scattering and reduced gloss of the film [29]. The results demonstrate that the film gloss is superior at a 5.0–15.0% concentration.

#### 3.3.3. Effect of Microcapsule Concentration on Mechanical Properties

The effect of microcapsules on the hardness, impact resistance and adhesion of the primer film is demonstrated in Table 5. With increasing microcapsule concentrations, the impact resistance increased, and the hardness increased from H to 4H. The dynamic loading capacity of the film surface was investigated by testing the impact resistance. The impact resistance of the film containing 0–15.0% microcapsules increased from 5.0 kg·cm to 10.0 kg·cm, and that of film with 30.0% microcapsules reached 12.0 kg·cm. The results show that the film had a good impact resistance, which was due to the favorable compatibility between the microcapsule particles and the film and the uniform distribution of microcapsules in the primer matrix. When the primer film was impacted, the impact stress acted on the wall material of the microcapsule particles and was quickly transmitted to the edge of the wall material; and the wall material had a certain toughness and compressive strength, so it could play a buffering role and reduce the internal stress of the matrix material, thereby ameliorating the impact resistance of the film to a certain extent [30]. Adhesion of the primer film is a precondition of a number of decorative and protective properties. It is an important index to evaluate the adhesion between the film and the base material. The test results showed that the adhesion grade of the film containing 0–20.0% microcapsule concentration was 0, and the adhesion performance was excellent, which indicated that the original superior adhesion of the primer film can be maintained by increasing the concentration of microcapsules in the primer. However, when the concentration was greater than 20.0%, the adhesion dropped to grade 1. This was because the concentration was too high, and the mechanical adhesive force between the film and the wood was slightly reduced, which affected the adhesion [31].

#### 3.3.4. Effect of Microcapsule Concentration on Liquid Resistance

The determination of the cold liquid resistance of the film on the wood surface served to investigate the antipollution performance of the film. For the primer film with 0–30.0% microcapsule concentration, a liquid resistance test of NaCl, detergent, ethanol and red ink was performed. The temperature was set at 16 °C, and the chromatic parameters of the film were measured before and after 24 h; the color difference is shown in Table 6. According to Table 7, the liquid resistance results of the film are shown in Table 8. The larger the color difference, the worse the liquid resistance; the lower the liquid resistance grade, the better the liquid resistance. After four kinds of liquid resistant solution tests, the color value of the red ink changed most apparently. The film with a 0–30.0% microcapsule concentration had a liquid resistance grade of 1 for sodium chloride and ethanol, and had no mark. As the concentration of microcapsules was 0–15.0%, the film liquid resistance to the detergent was better, i.e., grade 1. However, when the concentration of microcapsule was greater than 15.0%, the liquid resistance of the film to detergent decreased to grade 2, with slight discoloration marks. However, the liquid resistance of the film to red ink decreased when the concentration of microcapsules increased. These results indicate that red ink had more impact on the film than the other three liquids.

#### 3.3.5. Microstructure and Infrared Analysis of Films

**Figure 13:**
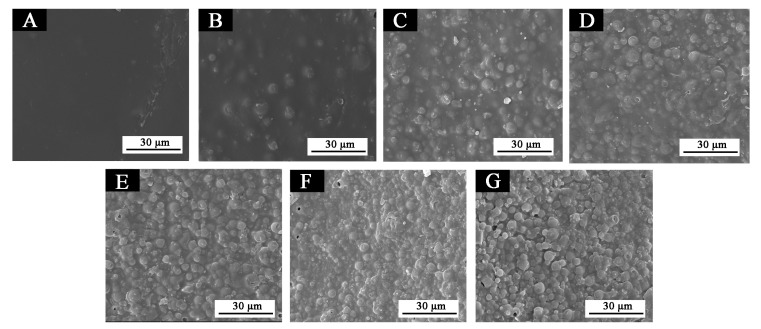
SEM of primer film containing different concentrations of thermochromic microcapsules: (**A**) 0%; (**B**) 5.0%; (**C**) 10.0%; (**D**) 15.0%; (**E**) 20.0%; (**F**) 25.0%; (**G**) 30.0%.

**Figure 14:**
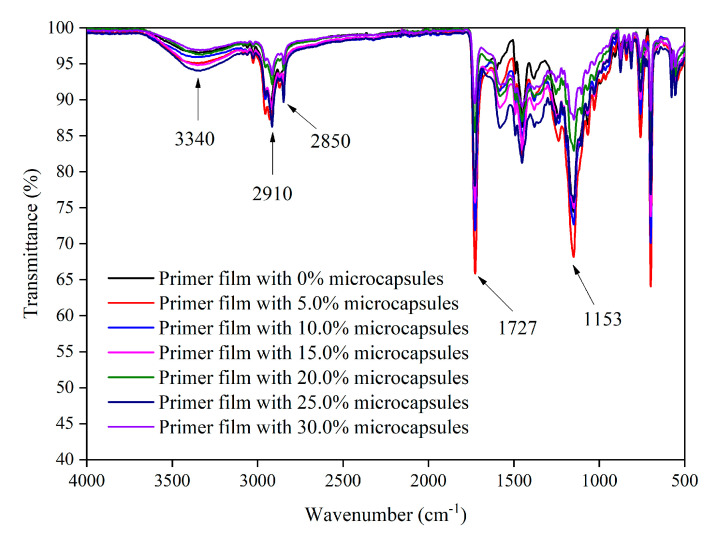
FTIR of primer film containing different concentrations of thermochromic microcapsules.

It is clear in Figure 13a that the film without microcapsules contained no particulate matter. When the concentration of microcapsules was 5.0–30.0%, the particles in Figure 13b–g were well-distributed without agglomeration, and the number of particles increased with the increase of microcapsules. These microcapsules uniformly dispersed in the primer film. Figure 14 shows the infrared spectrum of the primer film with different concentration of microcapsules. The stretching vibration absorption of –NH and –OH were superimposed at 3340 cm^−1^. It is known that 1153 cm^−1^ is the stretching vibration absorption of aromatic ether C–O–C; 1732 cm^−1^ was strong, along with sharp carbonyl characteristic absorption; 2910 cm^−1^ was a stretching vibration of –CH_3_. With the change of microcapsule concentration, no peaks disappeared or appeared, which demonstrated that there was no distinction in the component part of the primer film containing different concentrations of microcapsules. This indicated that no chemical reaction had occurred between the microcapsules and the primer film on Tilia europaea.

## 4. Conclusions

Our orthogonal experiments demonstrated that the concentration of microcapsules has the most obvious influence on the color difference of thermochromic film, compared with the temperature or drying time. With the concentration increasing, the discoloration temperature was 32 °C and changed from yellow to colorless. The color recovery temperature was 28 °C when the temperature was decreasing, and it reverted back to the original yellow color. As the concentration increased, the gloss of the film decreased and the hardness increased from H to 4H. The adhesion changed from grade 0 to 1, which was represented a minor decrease. The impact resistance increased from 5.0 kg·cm to 12.0 kg·cm. Furthermore, with an increase of concentration, the color difference of the primer film increased and the liquid resistance grade decreased. The overall performance of the film containing 15.0% microcapsules was the best. At this time, the film color difference was basically maximal, and the gloss at 60° was 10.6%, which constituted the subgloss. This film had better mechanical properties (i.e., adhesion of grade 0, hardness of 3H, impact resistance of 10.0 kg·cm). Except for red ink, which was grade 3, the resistance to other liquids was grade 1. The prepared waterborne primer film could be used as a decorative indicator material applied to indoor furniture surfaces to obtain temperature response.

## Figures and Tables

**Table 1:** Orthogonal experiment design.

Sample (#)	Microcapsules Concentration (%)	Drying Temperature (°C)	Drying Time (h)
1	15.0	35.0	2.0
2	15.0	60.0	4.0
3	30.0	35.0	4.0
4	30.0	60.0	2.0

**Table 2:** Ingredient of waterborne coating added with thermochromic microcapsules.

Sample (#)	Microcapsules Concentration (%)	Weight of Thermochromic Microcapsule (g)	Weight of Waterborne Primer (g)
1	15.0	15.0	85.0
2	15.0	15.0	85.0
3	30.0	30.0	70.0
4	30.0	30.0	70.0
5	0.0	0.0	100.0
6	5.0	5.0	95.0
7	10.0	10.0	90.0
8	15.0	15.0	85.0
9	20.0	20.0	80.0
10	25.0	25.0	75.0
11	30.0	30.0	70.0

**Table 3:** Analysis results of the orthogonal experiment.

Sample (#)	Microcapsules Concentration (%)	Drying Temperature (°C)	Drying Time (h)	The Color Difference of 16 °C and 32 °C in the Heating Process
1	15.0	35.0	2.0	68.5 ± 1.47
2	15.0	60.0	4.0	65.4 ± 1.18
3	30.0	35.0	4.0	73.9 ± 1.49
4	30.0	60.0	2.0	74.0 ± 2.45
Mean 1	66.950	71.200	-	-
Mean 2	73.950	69.700	-	-
Range	7.000	1.500	-	-

**Table 4:** Effect of microcapsule concentration on gloss.

Microcapsules Concentration (%)	20° Gloss (%)	60° Gloss (%)	85° Gloss (%)
0	8.60 ± 0.29	39.00 ± 1.02	51.20 ± 0.86
5.0	2.90 ± 0.08	10.60 ± 0.29	22.20 ± 0.67
10.0	1.80 ± 0.04	4.60 ± 0.16	10.20 ± 0.28
15.0	1.60 ± 0.04	3.10 ± 0.08	12.20 ± 0.22
20.0	1.70 ± 0.04	2.60 ± 0.08	10.90 ± 0.22
25.0	1.70 ± 0.03	2.50 ± 0.08	10.50 ± 0.41
30.0	1.70 ± 0.02	2.20 ± 0.08	11.20 ± 0.25

**Table 5:** Effect of microcapsule concentration on hardness, adhesion and impact resistance.

Microcapsules Concentration (%)	Hardness	Adhesion (Grade)	Impact Resistance (kg cm)
0	H ± 0	0 ± 0	5.00 ± 0.08
5.0	2H ± 0	0 ± 0	7.00 ± 0.14
10.0	3H ± 0	0 ± 0	7.00 ± 0.22
15.0	3H ± 0	0 ± 0	10.00 ± 0.29
20.0	3H ± 0	1 ± 0	11.00 ± 0.14
25.0	4H ± 0	1 ± 0	11.00 ± 0.41
30.0	4H ± 0	1 ± 0	12.00 ± 0.16

**Table 6:** Effect of microcapsule concentration on color difference of primer film before and after liquid resistance.

Microcapsules Concentration (%)	After the Test (Red Ink)	After the Test (NaCl Solution)	After the Test (Ethanol)	After the Test (Detergent)
0	6.40 ± 0.22	1.80 ± 0.04	0.80 ± 0	1.80 ± 0.05
5.0	21.70 ± 0.57	1.20 ± 0.03	3.00 ± 0.08	3.00 ± 0.08
10.0	26.90 ± 0.94	3.00 ± 0.08	2.50 ± 0.08	2.80 ± 0.08
15.0	35.70 ± 0.42	3.00 ± 0.08	2.70 ± 0.08	3.00 ± 0.08
20.0	42.50 ± 1.16	2.60 ± 0.08	2.30 ± 0.08	5.70 ± 0.16
25.0	60.40 ± 1.18	2.00 ± 0.08	2.50 ± 0.08	10.10 ± 0.29
30.0	63.20 ± 1.88	2.50 ± 0.08	3.00 ± 0.08	19.90 ± 0.70

**Table 7:** Classification of liquid resistance of furniture paint film.

Grade	Situation
1	No visual change (no damage).
2	Only when the light reaches the test surface or is very close to the mark and reflects to the eye of the observer, there is slight visible discoloration or discontinuous marks.
3	Slight impression, visible in several directions, such as a nearly complete ring or circle mark.
4	The surface structure has not changed significantly.
5	Severe effect, change of surface structure, tearing of surface material in whole or in part, or adhesion of paper to test surface.

**Table 8:** Effect of microcapsule concentration on liquid resistance.

Microcapsules Concentration (%)	Red Ink	NaCl Solution	Ethanol	Detergent
0	2 ± 0	1 ± 0	1 ± 0	1 ± 0
5.0	3 ± 0	1 ± 0	1 ± 0	1 ± 0
10.0	3 ± 0	1 ± 0	1 ± 0	1 ± 0
15.0	3 ± 0	1 ± 0	1 ± 0	1 ± 0
20.0	4 ± 0	1 ± 0	1 ± 0	2 ± 0
25.0	4 ± 0	1 ± 0	1 ± 0	2 ± 0
30.0	4 ± 0	1 ± 0	1 ± 0	2 ± 0
